# Effects of Surface Treatment with Thymol on the Lipid Oxidation Processes, Fatty Acid Profile and Color of Sliced Salami during Refrigerated Storage

**DOI:** 10.3390/foods11233917

**Published:** 2022-12-05

**Authors:** Éva Varga-Visi, Ildikó Jócsák, Vanda Kozma, Katalin Lóki, Omeralfaroug Ali, András Szabó

**Affiliations:** 1 Department of Physiology and Animal Health, Institute of Physiology and Nutrition, Kaposvár Campus, Hungarian University of Agriculture and Life Sciences, Guba Sándor Street 40, H-7400 Kaposvár, Hungary; 2 Department of Agronomy, Institute of Agronomy, Kaposvár Campus, Hungarian University of Agriculture and Life Sciences, Guba Sándor Street 40, H-7400 Kaposvár, Hungary; 3 Kaposvár Campus, Hungarian University of Agriculture and Life Sciences, Guba Sándor Street 40, H-7400 Kaposvár, Hungary; 4 Department of Chemistry, Institute of Mathematics and Basic Science, Kaposvár Campus, Hungarian University of Agriculture and Life Sciences, Guba Sándor Street 40, H-7400 Kaposvár, Hungary; 5 Agribiotechnology and Precision Breeding for Food Security National Laboratory, Department of Physiology and Animal Health, Institute of Physiology and Nutrition, Hungarian University of Agriculture and Life Sciences, Guba Sándor Street 40, H-7400 Kaposvár, Hungary

**Keywords:** fatty acid composition, oxidative deterioration, thiobarbituric acid-reactive substances, meat color, metmyoglobin, paprika, thymol, antioxidant

## Abstract

The oxidation of unsaturated fatty acids and the adverse transformation of pigments from meat and spices are the primary causes of chemical degradation in processed meat products. Thymol is found in a variety of plant extracts that have been proven to effectively inhibit or slow down oxidative processes. The objective of our study was to determine whether thymol treatment of the surface of sliced paprika salami could be applied to inhibit lipid oxidation and color change during refrigerated storage. During eight weeks of storage, the malondialdehyde (MDA) levels and the ratios of saturated fatty acids (SFAs), monounsaturated fatty acids (MUFAs), polyunsaturated fatty acids (PUFAs), and n6/n3 in thymol-treated salami remained unchanged (*p* ≥ 0.05), whereas in the controls, the MDA levels increased by approximately twelvefold and the ratio of SFAs in the lipid fraction increased (*p* < 0.001), while the ratio of PUFAs decreased (*p* < 0.001). The application of thymol prevented decrease in yellowness (b*) of the slices and reduced decreases in redness (a*) and brightness (chroma).

## 1. Introduction

The vast majority of meat products contain significant amounts of lipids. Lipids are essential for the development of desirable organoleptic properties in meat products, influencing the formation of the ideal aromas, tastes, and textures. Moreover, lipids play an important role in human nutrition due to their nutritional value and particular functions. In addition to providing energy for biological processes, lipids serve a multitude of other physiological functions and contain components, such as essential fatty acids and fat-soluble vitamins, that cannot be synthesized endogenously in humans and animals and which therefore must be obtained from the diet [1,2]. However, the transformation of lipids, specifically the oxidation of unsaturated fatty acids (FAs), is frequently a significant factor in decline in food quality. In addition to microbiological processes, the rate of oxidative degradation of lipids ultimately determines the shelf life of the majority of processed meat products. Oxidative processes degrade not only the organoleptic properties of products but may also result in significant nutrient losses and the production of toxic substances, making their control a crucial task for the meat industry [1,2,3,4].

Nowadays, consumers increasingly expect food manufacturers to use natural substances and, as a consequence, meat-product manufacturers are also increasingly seeking to replace synthetic antioxidants with natural substances or extracts whose active antioxidant ingredients are able to prevent or delay oxidative degradation [5,6,7]. However, the active contents of natural substances and extracts are affected by a number of factors. The active ingredient contents of a plant depend on genetic variation within the species, agrotechnical practices, the season, the geographic origin of the plant and the plant part used (e.g., root, seed or leaves). The active ingredient contents of food additives or extracts derived from plants are highly dependent on the technology used in the production/extraction, e.g., on whether distillation or solvent extraction was used, as well as the plant part from which the extract was taken [8,9,10,11]. It follows from the above that for food additives with antioxidant activities derived from plants or plant extracts, great emphasis should be placed on standardizing the levels of active substances, while another possibility is to use active substances directly to ensure oxidative stability.

Oregano and thyme are commonly used in meat products, having significant antioxidant activity in addition to their pleasant organoleptic properties [5,6]. Both herbs contain substantial amounts of thymol (2-isopropyl-5-methylphenol or iso-propylmetha-cresol; C_10_H_14_O), a monoterpene phenolic compound with antioxidant properties. When added to a variety of food ingredients/meat types (such as fish, chicken, veal, beef, lamb and pork), thyme extract and essential oil have been shown to inhibit or delay oxidative processes [10].

Its positional isomer, carvacrol, is always present alongside thymol in plants of the *Labiatae* family, including thyme. Both compounds are important antioxidant components of thyme essential oil [10]. Due to the different positions of the phenolic group in the two isomers, thymol has a greater steric hindrance than carvacrol. Thymol proved to be a more potent antioxidant than carvacrol with respect to lard triglycerides and sunflower oil [12]. *Thymus zygis* subsp. *gracilis* leaves contain between 22.3 and 43.3% thymol, whereas the leaves of *Thymus vulgaris* contain 38.1% thymol [13,14]. Thymol makes up 40–80% of the phenolic compounds in thyme essential oil. This value may vary depending on the thyme species and geographical origin. As the thymol contents of plant-based extracts and thus the extent of the antioxidant effects they exert are influenced by a number of factors, it is reasonable to deduce that it would be beneficial to investigate the antioxidant effects of pure thymol on meat products. Furthermore, if its effect is favorable and significant, the application of thymol would be more reproducible than that of herbal materials and products the composition of which may be affected by uncontrollable factors. However, before such a study can be conducted, it is necessary to determine whether the direct use of thymol is safe for human consumption. The Food and Drug Administration (FDA) of the United States classifies thymol as GRAS (generally recognized as safe). Several essential oil components, including thymol, have been approved by the European Commission for use in food flavoring because they do not pose risks to consumer health [10,15,16]; therefore, their use in food preservation is permitted.

Thymol can provide consumers with health benefits in addition to preserving food. Historically, thymol-containing plants have been utilized in traditional medicine [17]. In general, the biologically active constituents of herbs with anti-tumor, anti-inflammatory and anti-cancer properties are also antioxidant compounds, and their health benefits are associated with reduced oxidative stress. Thymol exerts anti-inflammatory and anti-tumor effects, and it has been used to treat circulatory, nervous and digestive disorders [10,17,18]. Consumers frequently consume meat products with limited shelf lives in multiple batches rather than immediately, storing them in their refrigerators after opening the packaging. However, if the integrity of the packaging is compromised, its protective effect is diminished or eliminated, and the food may spoil. Utilizing preservation techniques that safeguard the quality of food under these conditions leads to reduction in this type of food waste. In order to accomplish such preservation, we investigated whether thymol treatment of the surface of sliced salami could be used to inhibit oxidative rancidity under oxidative conditions. To monitor the oxidative processes, the color coordinates of the salami surfaces were determined, and the amounts of malondialdehyde (MDA)-like substances produced during lipid oxidation, as well as the fatty acid compositions of the lipids, were measured.

## 2. Materials and Methods

The tests were carried out on a dry-fermented meat product, a paprika salami, purchased from a local producer in Kaposvár, Hungary. Paprika (dried and ground red pepper (*Capsicum* sp.) and oleoresins are frequently used ingredients in sausages in Hungary. The ingredients of the salami were: pork (130 g of pork per 100 g of product), back fat, ground sweet pepper, sodium chloride, sodium glutamate, sodium ascorbate, sodium nitrite, sugars (sucrose and glucose) and starter culture. According to the basic compositional data provided by the manufacturer, 100 g of finished product contained 37 g of fat, including 15 g of saturated fatty acids. The protein content was 21 (*w/w*)%, the carbohydrate content was 1.5% (of which 0.7% sugar) and the product contained 3.7% sodium chloride.

For the storage experiment, the samples were prepared as follows. After removal of the coating, the salami was sliced using a Bosch universal slicer (Bosch GmbH, Gerlingen, Germany). The diameter of the slices was 45 mm, and the weight of the slices was approximately 8 g. On the surface of each slice, 0.2 cm^3^ of thymol solution was applied. Ethanol was used to dissolve thymol, which can be regarded as a bio-derived solvent [19]. The concentration of the solution was 20 mg thymol/cm^3^ ethanol; the volume that was applied to the surface of each salami slice represented 4 mg of thymol per slice and 500 mg thymol per kg salami. Only the solvent was applied to the control salami slices at the same volume (0.2 cm^3^ ethanol per slice). The solvent was evaporated from the slices under a chemical fume hood at room temperature, and the slices were placed in plastic storage boxes. Two slices of treated and two slices of control salami were placed in a plastic box 14 × 14 cm wide and 5.5 cm high with a lockable lid and equipped with a rigid mesh with a perforated structure between them, in order to prevent contact but to allow the thymol-treated and control samples to be in the same air space. One box was considered as an experimental unit. Six experimental units were stored refrigerated (6 ± 1 °C) for eight weeks; therefore, the number of repetitions was six. The boxes were opened once a week for five minutes and the CIELAB color coordinates of the surfaces of the slices were determined [20], and the boxes were then reclosed and further stored. That is, instrumental color parameters were measured weekly for each experimental unit containing both control and treated salami samples. Fatty acid compositions and amounts of malondialdehyde (MDA) equivalents were determined at the beginning of the storage and at the end of the storage. That is, fatty acid compositions and MDA levels were measured in fresh samples before the start of storage, directly after the slicing of the salami, to assess the initial stage, and also at the end of the eight-week storage, when all of the contents of the experimental units were used up for FA and MDA analysis.

Instrumental color parameter and MDA analyses were conducted as described elsewhere [21,22]. Prior to fatty acid analysis, the samples were homogenized (IKA T25 ultra turrax, IKA, Staufen, Germany) in a 20-fold volume of chloroform:methanol (2:1 *vol:vol*), containing 100 mg/L butylated hydroxytoluene (BHT) as an antioxidant along with an internal standard (C19:0, Sigma, cat. no. 72332, Supelco, Bellefonte, PA, USA). Total lipids were extracted from the homogenized samples using the method of Folch et al. [23]. Fatty acid transmethylation was carried out with 1% H_2_SO_4_ in methanol, according to Christie [24]. Fatty acid composition was determined via gas chromatography (Shimadzu Nexis 2030, Kyoto, Japan) in the form of fatty acid methyl esters (FAMEs), after separation in a Zebron ZB-WaxPlus capillary column (30 m × 0.25 mm × 0.25 micrometer film, Phenomenex Inc., Torrance, CA, USA). The chromatographic evaluation was performed with LabSolutions 5.93 software, using the PostRun module (Shimadzu, Kyoto, Japan) with manual peak integration. Fatty acid composition was expressed as weight % of total FAMEs. The identification of fatty acids was performed based on the retention time of a CRM external standard (Supelco 37 Component FAME Mix, Merck-Sigma Aldrich, CRM47885, Steinheim, Germany). The identification of thymol was based on the use of an external standard (Sigma, cat. no. PHR1134, Supelco, Bellefonte, PA, USA.)

One-way analysis of variance was used to compare MDA levels and fatty acid compositions among groups (control fresh, control stored and treated stored). The effects of thymol treatment and storage period (as factors) on color parameters were analyzed with two-factor analysis of variance. In case of significant differences among treatment averages, pairwise comparison was performed with the Student–Newman–Keuls test. In case of a significant difference in group variance, the Tamhane test was applied. The pre-set significance level (*p*-value) used for statistical decisions was 0.05. Statistical analysis was performed using IBM SPSS Statistics 20.0 (2010).

## 3. Results

### 3.1. Effects of Thymol Treatment and Storage on MDA Levels

**Figure 1:**
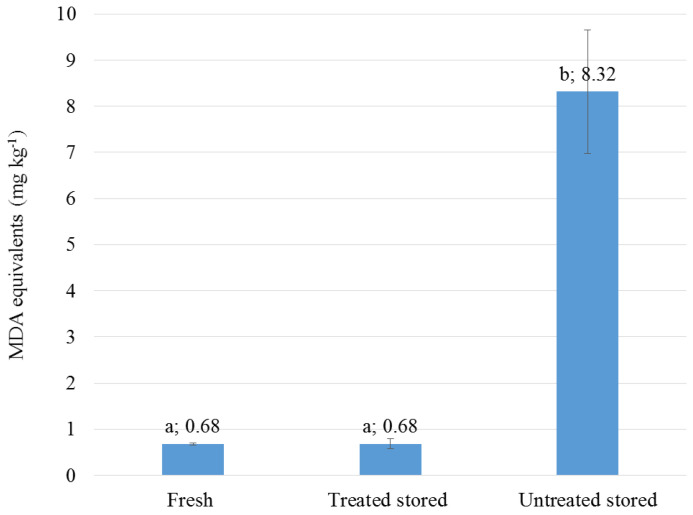
The effect of treatment with thymol on the oxidative stability of the sausage samples. Stored samples were refrigerated at 6 °C for eight weeks. Significant differences (*p* < 0.05) between the mean values (*n* = 6) are indicated by different letters at the top of the columns.

As shown in Figure 1, the average MDA levels of the samples treated with thymol and stored refrigerated for eight weeks (treated stored) were practically the same as the levels measured at the beginning of storage (fresh). In contrast, the MDA levels of the samples not treated with thymol increased to about twelve times the initial values by the end of storage (untreated stored).

### 3.2. Effects of Thymol Treatment and Storage on Fatty Acid Profiles

**Figure 2:**
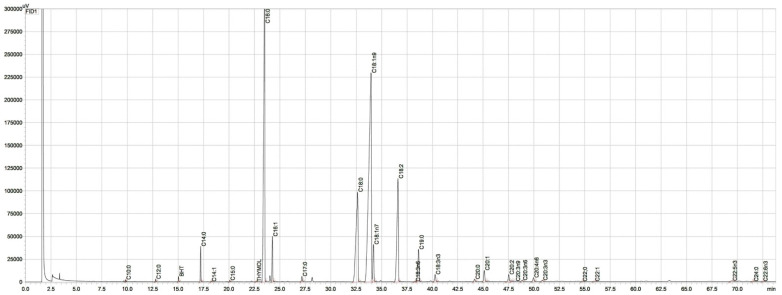
Chromatogram of a sausage sample with abbreviations of the identified fatty acids. Fatty acids were separated in the form of fatty acid methyl esters (FAMEs). Butylated hydroxytoluene (BHT) was added during extraction to avoid the further oxidation of fatty acids during analytical sample preparation (retention time: 15 min). The internal standard was C19:0. The retention time of thymol was 22.6 min.

The analysis of fatty acid compositions revealed 26 individual and identified fatty acids (Figure 2, Table 1). Palmitic acid (C16:0) and stearic acid (C18:0) accounted for 94–95% of the saturated fatty acid (SFA) fractions in the lipids of the salami samples (Table 1). Among the monounsaturated fatty acids (MUFAs), oleic acid (C18:1 n9) comprised the majority (86–87%), whereas linoleic acid (C18:2) comprised the majority (86–88%) of the polyunsaturated fatty acid (PUFA) fractions.

At the end of refrigerated storage, the untreated samples (untreated stored, Table 1) contained marginally more palmitic acid (C16:0) and stearic acid (C18:0) than the samples treated with thymol (treated stored; Table 1). However, for oleic acid (C18:1 n-9), no significant difference was observed between untreated and treated samples after 8 weeks. Furthermore, there was a general tendency for polyunsaturated fatty acids to be present in a higher proportion in the lipids of the thymol-treated samples than in the lipids of the control samples. The thymol-treated stored samples contained higher proportions of the following PUFAs than the untreated stored samples: linoleic acid (C18:2 n-6), alpha-linolenic acid (C18:3 n-3), eicosadienoic acid (C20:2 n-6), eicosatrienoic acid positional isomers (C20:3 n-9, C20:3 n-6 and C20:3 n-3), arachidonic acid (C20:4 n-6) and docosapentaenoic acid (C22:5 n-3).

The untreated samples’ margaric acid (C17:0), stearic acid (C18:0) and docosenoic acid (C22:1n-9) lipid fraction proportions increased significantly during storage. In contrast, linoleic acid, alpha-linolenic acid, eicosatrienoic acid positional isomers of C20:3 n-6 and C20:3 n-3, arachidonic acid (C20:4 n-6) and docosapentaenoic acid proportions decreased significantly (Table 1).

Small but significant increases in the ratios of stearic acid (C18:0), eicosadienoic acid (C20:2 n-6) and eicosatrienoic acid (C20:3 n-9, mead acid) were observed in the thymol-treated samples during storage, while the lipid contents of the other fatty acids did not significantly change.

It can be seen that the fatty acid compositions of the untreated samples were significantly altered, while those of the treated samples changed only minimally during cold storage. The same conclusion can be drawn when comparing the values obtained before and after storage for the fatty acid groups of SFAs, MUFAs and PUFAs (Table 1). The proportions of these fatty acid fractions in the treated stored samples were the same as in the fresh samples, whereas in the untreated stored samples the proportions of SFAs were greater and the proportions of PUFAs were smaller than those of the fresh samples.

According to Table 1, both n-3 and n-6 fatty acid levels were lower in the untreated stored samples than in the fresh samples. That is, both levels decreased during storage in the untreated samples. However, these decreases had a greater impact on n-3 fatty acids than on n-6 fatty acids, as the n-6/n-3 ratios were higher in the untreated stored samples than in the fresh samples.

### 3.3. Effects of Thymol Treatment and Storage on the Surface Color Coordinates of the Salami Slices

**Figure 3:**
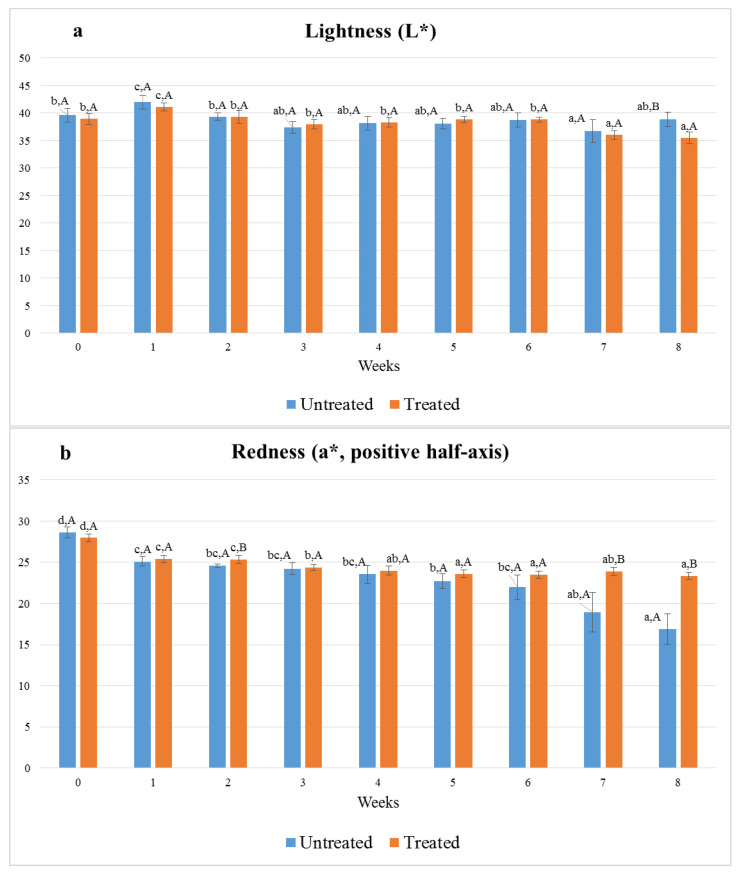

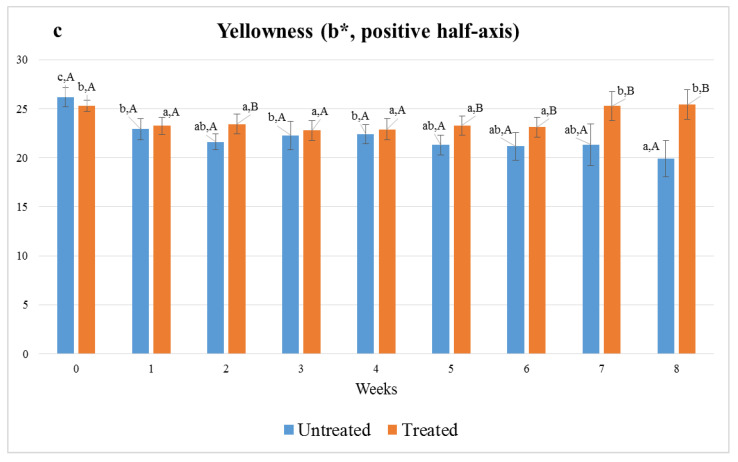
The effect of treatment with thymol on color coordinates. The means of lightness (L*), (**a**) redness (a*) (**b**) and yellowness (b*) (**c**) values of sausages refrigerated at 6 °C for eight weeks. Lowercase superscript letters show the effects of time. Capital letter superscripts show the effects of treatment. Means sharing common superscripts did not differ significantly (*p* > 0.05).

The CIELAB color coordinates (L*, a* and b*) of the samples were determined weekly. The first measurements were performed before the start of the storage (0 weeks; Figure 3). As shown in Figure 3a, the luminosity or lightness (L*) levels of the thymol-treated salami slices and those not treated with thymol did not differ significantly until the seventh week of storage. Untreated samples were slightly brighter than treated ones at the eighth week; the means for L* were 38.9 and 35.5, respectively. The surface brightness levels of the treated and untreated samples showed similar fluctuations over time.

The measured values for the red–green color coordinate (a*) fell on the positive semi-axis; consequently, their absolute values were attributable to the redness of the color. The redness of the surface of the salami slices decreased as a function of cold storage duration (Figure 3b), but the decrease in a* was more pronounced for the untreated samples. During the seventh and eighth weeks of storage, the surface a* values of the thymol-treated samples were significantly greater than those of the untreated samples at the respective sampling timepoints.

The values of the yellow–blue color coordinate (b*) fell on the positive semi-axis; consequently, their absolute values were proportional to the yellowness of the color. As depicted in Figure 3c, the yellowness of the untreated samples decreased overall as compared to the treated samples. From the fifth week of storage until the end of it, the surface b* values of the treated samples were significantly higher than those of the untreated samples.

**Figure 4:**
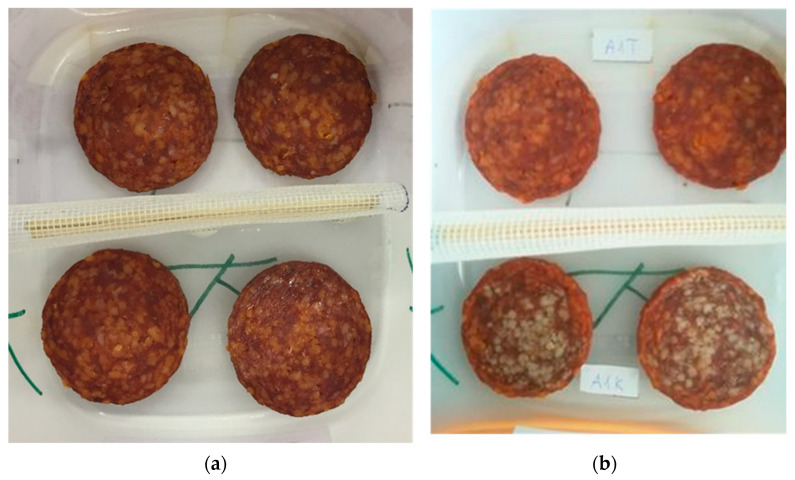
Sausage samples in the storage box at the beginning (**a**) and at the end (**b**) of the eight-week refrigerated storage (6 °C). Thymol-treated slices are in the upper parts, while untreated slices are in the lower parts of the photos.

Figure 4 depicts an experimental unit, a box with four slices of salami, at the beginning of storage (Figure 4a) and after eight weeks of refrigerated storage (Figure 4b). Separated by a net, the box contained two slices of salami treated with thymol and two slices of untreated salami. The top two slices in the image were treated with thymol, while the bottom two were untreated. At the beginning (Figure 4a) and end of storage (Figure 4b), the colors of the thymol-treated slices were nearly identical, whereas significant portions of the surfaces of the untreated slices were discolored.

The values for chroma (C*) and hue angle (h_ab_) are displayed in Table 2. Chroma gradually decreased during the eight-week storage, but this decrease was significantly lesser in the salami slices treated with thymol (from 37.7 to 34.5) than in the slices not treated with thymol (from 38.8 to 26.1). That is, the color of the slices faded over time, changed from vivid to pale, but the loss of color intensity was partly inhibited by thymol. During storage, the hue angle values increased, while the untreated samples exhibited little to no difference from the treated samples at the same time points. Overall, the changes in chroma were more pronounced than the changes in hue angle.

## 4. Discussion

Chemical decomposition processes in meat products result in alterations that make them unsuitable for consumption and may even endanger consumers’ health. During refrigerated storage, the oxidation of pigments causes undesirable changes in surface color, and secondary processes of lipid oxidation produce unpleasant odors and flavors; some of the products of lipid oxidation may also exert negative health effects [1,2,4]. In addition to microbial processes, the safety, quality and shelf life of meat products are determined by those associated with lipid oxidation and pigment deterioration.

Our results indicated that the applied surface treatment with thymol was able to prevent the lipid oxidation of sliced salami with paprika until the end of the cold storage period, as it hampered the formation of MDA-like substances which are the products of secondary processes of lipid oxidation [2] at the dose applied. Moreover, thymol treatment was able to conserve the initial fatty acid profile of the product.

### 4.1. Fatty Acid Profiles

The increase in the proportion of SFAs in the untreated samples occurred at the expense of PUFAs, while the MUFA proportions remained unchanged. Significant decomposition of fatty acids with two or more double bonds was observed, whereas no change was generally observed for those with one double bond. This observation is consistent with the fact that PUFAs have bisallylic methylene position(s) and that the methylene group in this structure has a significantly lower carbon–hydrogen dissociation energy than the methylene group adjacent to the allyl group in MUFAs. For example, linoleic acid undergoes oxidation at a rate approximately ten times faster than that of oleic acid [1,4,25]. On the basis of the measured MDA levels, the oxidative degradation of the salami slices stored for eight weeks without treatment was extremely advanced. In line with this, we did not observe a significant decrease in the oleic acid proportions of lipids, whereas the linoleic acid proportions decreased significantly.

In the untreated samples, the total n-3 FA proportions decreased to a greater extent than those of n-6 fatty acids, leading to more unfavorable, increased n-6/n-3 ratios during storage. These results were consistent with the observation that the rate of oxidation also depends on the positions of the methylene-interrupted double bonds in the alkyl chain, as n-3 fatty acids are oxidized faster than n-6 fatty acids [25]. Similarly, during 12 months of cold storage of Iberian chorizo salami in modified-atmosphere packaging, a decrease in PUFA levels and increases in SFA and n6/n3 ratios were observed [26].

### 4.2. Lipid Peroxidation/Malondiadehyde

Meat undergoes simultaneous oxidation of pigments and unsaturated fatty acids, but these two processes promote and reinforce each other via their intermediates. Certain unsaturated aldehyde breakdown products of the hydroperoxides formed from unsaturated fatty acids, for instance, 4-hydroxynonenal, promote the conversion of oxymyoglobin to metmyoglobin [2,27]. During the oxidation of oxymyoglobin to metmyoglobin, reactive oxygen species and an activated metmyoglobin complex are produced, which promote the oxidation of unsaturated fatty acids. In a previous study, a negative correlation (*p* ˂ 0.001) was found between MDA levels and a*, b* color coordinates during storage of sliced paprika salami [22]. In the current study, an increase in MDA levels (and a decrease in PUFA ratios) was associated with a decrease in a* and b* values in the untreated samples. Together with the oxidation of unsaturated fatty acids, the transformation of chromophore compounds decreased the redness and yellowish hue of the surface of the salami samples.

### 4.3. Surface Color and Oxidative Damage

During the storage of the salami slices under oxidative conditions, the surface redness (a*) and yellowness (b*) of the thymol-treated slices changed less than they did in the control slices. The decrease in a* and b* may be attributable to a decrease in the level of the meat pigments nitrosylmyoglobin and oxymyoglobin, accompanied by an increase in metmyoglobin levels [28]. Oxymyoglobin had a pro-oxidant effect on unsaturated lipids under the oxidative conditions used in our study, while being oxidized to brown-colored metmyoglobin [29]. However, when nitrite salt is added to salami, a significant proportion of hem pigments are present as nitrosylmyoglobin (“cured meat pigment”). Nitrosylmyoglobin is a stable compound in the absence of oxygen, but in the presence of oxygen or other oxidizing agents, such as peroxides resulting from lipid oxidation, it is converted to metmyoglobin through the formation of nitrate or nitrite. Nitrosylmyoglobin depletes oxygen or peroxides during this process and therefore acts as an antioxidant in this respect, but it is used up and its end product is also the brown-colored metmyoglobin, similar to that of pro-oxidant oxymyoglobin [28,29,30,31]. The discoloration that occurs during oxidation has a significant effect on the visual appeal of meat products. In the development of stable color in commercial meat products, the formation of metmyoglobin poses the greatest challenge [28]. The thymol treatment in our study was able to significantly reduce the degree of color change in salami slices under oxidative conditions, presumably due to the effective inhibition of lipid peroxidation, as proven by the MDA results.

In addition to the oxidation of chromophore compounds in the meat, the oxidation of pigments in the dried and ground red pepper (paprika) could also affect the color coordinates, as the carotenoid compounds that give ground pepper its characteristic color are also susceptible to oxidation [32]. During refrigerated storage of sliced sausage containing dried ground pepper, a reduction in redness and/or yellowing was also observed. Garcia-Torres and coworkers [26] discovered a decline in both indices when refrigerated Iberian chorizo was packaged in a modified atmosphere for a year. Pereira and coworkers [33] (2019) observed a reduction in a* values only when vacuum-packed Iberian chorizo was kept under artificial lighting for six months.

The manufacture of paprika affects the redness of the color (a*), as a high color stability was observed when using ground smoked paprika and a decrease in a* was observed when using ground sun-dried or artificially dried paprika [33]. The color-preserving and lipid-oxidation-inhibiting effects of smoked paprika powder were attributed to the antioxidant effects of phenolic compounds that originated from smoke. In our study, it was found that the phenolic antioxidant compound used to treat the surface of salami was also effective in inhibiting lipid oxidation and delaying undesirable color change.

The thymol surface treatment inhibited lipid oxidation until the end of the storage period, as there was no increase in MDA levels and the lipid fractions (SFAs, MUFAs and PUFAs) remained unchanged. Luna and coworkers [34], in a study on poultry feed (broiler feed mash), using thymol at a similar dose (400 mg/kg) to that used in our study, observed no change in PUFA or SFA ratios over a 60-day storage period, whereas in the control they observed a decrease in PUFA ratios and an increase in SFA ratios. Mastromatteo and coworkers [35] added 500 mg/kg of various plant-based active ingredients (thymol, eugenol and D-limonene) and essential oils (sweet lemon, orange, grapefruit and mandarin orange) to low-fat sausages. The organoleptic properties of thymol were found to be comparable to those of D-limonene and essential oils but significantly superior to those of eugenol. According to the authors, thymol was found to be the most desirable additive besides sweet lemon essential oil in tests of shelf life. On frankfurters, phytochemical-fortified chitosan coatings were applied [36]. All types of coatings resulted in lower TBARS values than those of the control during refrigerated storage. However, the TBARS values of sausages coated with thymol in chitosan were identical to those of sausages coated with chitosan alone. Using thymol and other essential oil components, a nanocomposite film based on linear low-density polyethylene was formed in order to package a traditional Turkish fermented sausage, *sucuk* [37]. When sliced sausages were stored at 10 °C for 20 days, packaging materials containing thymol inhibited lipid oxidation. The use of thyme essential oil in a protein coating improved the storage quality of *sucuk* [38]. Using this essential oil, the decrease in redness (a*) and yellowness (b*) and the increase in TBARS values were slowed during refrigerated storage, i.e., thyme oil was able to retard color change and lipid oxidation. Based on the preceding observations, thymol alone or in combination with other compounds appears to be a potent antioxidant for processed and stored muscle foods under various conditions.

Although thymol is generally recognized as safe (GRAS) and has been approved for food industrial use, the concentration at which it is considered to be safe for use has not yet been defined [16]. However, there may be instances when the amount of ingested thymol as an additive is significantly higher than the amount of thymol ingested in the diet and which is deemed safe based on long-standing experience. We used 4 mg of thymol per salami slice, which corresponds to the thymol content of approximately 0.7–0.8 deciliters of thyme tea [39]. If we ignore the evaporation and degradation of thymol during storage, then consuming 7 to 10 slices of salami treated with thymol would provide the same amount of thymol as drinking 2 to 3 cups of thyme tea. We have therefore been successful in inhibiting oxidative degradation with a dose of thymol that can be suitable for human consumption in normal dietary practices.

## 5. Conclusions

Based on the results of this preliminary work, the applied surface treatment with thymol proved to be an effective tool to hamper lipid oxidation in sliced salami under refrigerated conditions; furthermore, it efficiently postponed undesirable color changes on the surfaces of the slices. Nevertheless, before the application of this technique for food industrial purposes, further investigations are needed regarding the quality and safety of thymol-treated products. Specifically, the effect of thymol on the microbiological properties of the sausage has to be monitored during the storage process; moreover, organoleptic tests have to be conducted in order to evaluate the consumer acceptability of the product.

## Figures and Tables

**Table 1:** The effect of thymol treatment on the fatty acid compositions of the sausage samples, expressed as fatty acid methyl ester %. Stored samples were refrigerated at 6 °C for eight weeks.

Fatty Acid	Fresh	Untreated Stored	Treated Stored	SE	Sig.
C10:0	0.05	0.04	0.05	0.001	n.s.
C12:0	0.07	0.07	0.08	0.001	n.s.
C14:0	1.31	1.32	1.35	0.012	n.s
C14:1 n-5	0.02	0.02	0.02	0.000	n.s
C15:0	0.07	0.07	0.07	0.001	n.s.
C16:0	25.68 ^a,b^	25.99 ^b^	25.37 ^a^	0.088	*p* < 0.01
C16:1 n-7	2.47	2.45	2.42	0.010	n.s.
C17:0	0.36 ^a^	0.37 ^b^	0.36 ^a^	0.001	*p* < 0.05
C18:0	12.26 ^a^	12.63 ^c^	12.41 ^b^	0.043	*p* < 0.001
C18:1 n-9	39.88	40.05	39.85	0.042	n.s.
C18:1 n-7	2.88	2.89	2.87	0.004	n.s.
C18:2 n-6	12.03 ^b^	11.38 ^a^	12.16 ^b^	0.088	*p* < 0.001
C18:3 n-6	0.03	0.03	0.03	0.001	n.s.
C18:3 n-3	0.65 ^b^	0.57 ^a^	0.65 ^b^	0.010	*p* < 0.001
C20:0	0.18	0.18	0.18	0.001	n.s.
C20:1 n-9	0.76	0.77	0.78	0.005	n.s.
C20:2 n-6	0.49 ^a^	0.48 ^a^	0.53 ^b^	0.007	*p* < 0.001
C20:3 n-9	0.03 ^a^	0.03 ^a^	0.04 ^b^	0.001	*p* < 0.01
C20:3 n-6	0.11 ^b^	0.09 ^a^	0.11 ^b^	0.002	*p* < 0.001
C20:4 n-6	0.37 ^b^	0.29 ^a^	0.37 ^b^	0.010	*p* < 0.001
C20:3 n-3	0.10 ^b^	0.09 ^a^	0.10 ^b^	0.002	*p* < 0.001
C22:0	0.02	0.01	0.02	0.000	n.s.
C22:1 n-9	0.02 ^a^	0.04 ^b^	0.02 ^a^	0.002	*p* < 0.001
C22:5 n-3	0.08 ^b^	0.06 ^a^	0.09 ^b^	0.003	*p* < 0.001
C24:0	0.08	0.07	0.07	0.001	n.s.
C22:6 n-3	0.01	0.02	0.02	0.001	n.s.
SFAs	40.07 ^a^	40.76 ^b^	39.95 ^a^	0.102	*p* < 0.001
MUFAs	46.02 ^a,b^	46.21 ^b^	45.96 ^a^	0.043	*p* < 0.05
PUFAs	13.91 ^b^	13.03 ^a^	14.10 ^b^	0.119	*p* < 0.001
n-3	0.85 ^b^	0.73 ^a^	0.86 ^b^	0.014	*p* < 0.001
n-6	12.54 ^b^	11.79 ^a^	12.67 ^b^	0.100	*p* < 0.001
n-6/n-3	14.81 ^a^	16.07 ^b^	14.81 ^a^	0.154	*p* < 0.001

^a,b,c^ Means in a row sharing common letter indices are not different (*p* ≥ 0.05). n.s. = Null hypothesis of ANOVA was accepted, as there were no significant differences between means (*p* ≥ 0.05). SFAs = saturated fatty acids; MUFAs = monounsaturated fatty acids; PUFAs = polyunsaturated fatty acids.

**Table 2:** The effects of thymol treatment on the chroma (C*) and hue angle (h_ab_) values of the sausages refrigerated at 6 °C for eight weeks.

C*										
Storage (week)	0	1	2	3	4	5	6	7	8	SE
U	38.8 ^e,A^	34.0 ^d,A^	32.8 ^c,d,A^	32.9 ^c,d,A^	32.5 ^c,d,A^	31.1 ^c,A^	30.5 ^b,c,A^	28.6 ^b,A^	26.1 ^a,A^	0.51
T	37.7 ^c,A^	34.4 ^a,b,A^	34.5 ^a,b,B^	33.4 ^a,b,A^	33.2 ^a,b,A^	33.2 ^a,b,B^	33.0 ^a,B^	34.8 ^b,B^	34.5 ^a,b,B^	0.23
h_ab_										
Storage (week)	0	1	2	3	4	5	6	7	8	SE
U	42.5 ^a,b,A^	42.4 ^a,b,A^	41.3 ^a,A^	42.6 ^a,b,A^	43.6 ^a,b,A^	43.2 ^a,b,A^	43.9 ^b,A^	48.4 ^c,A^	49.7 ^c,B^	0.42
T	42.1 ^a,A^	42.4 ^a,A^	42.7 ^a,B^	43.0 ^a,b,A^	43.6 ^a,b,A^	44.6 ^b,B^	44.5 ^b,A^	46.6 ^c,A^	47.4 ^c,A^	0.28

^a,b,c,d,e^ Means in a row sharing common lowercase indices are not different (*p* ≥ 0.05). ^A,B^ Means in a column where one uppercase index is common are not different (*p* ≥ 0.05). U = untreated; T = treated.

## Data Availability

The data are available from the corresponding author on request.
